# A Detection Approach for Wheat Spike Recognition and Counting Based on UAV Images and Improved Faster R-CNN

**DOI:** 10.3390/plants14162475

**Published:** 2025-08-09

**Authors:** Donglin Wang, Longfei Shi, Huiqing Yin, Yuhan Cheng, Shaobo Liu, Siyu Wu, Guangguang Yang, Qinge Dong, Jiankun Ge, Yanbin Li

**Affiliations:** 1 College of Water Conservancy, North China University of Water Resources and Electric Power, Zhengzhou 450000, China; wangdonglin@ncwu.edu.cn (D.W.); chengyvhan6714@163.com (Y.C.); gejiankun@ncwu.edu.cn (J.G.); 2 School of Water Resources and Environment Engineering, Nanyang Normal University, Nanyang 473061, China; 3 College of Water Resources and Civil Engineering, China Agricultural University, Beijing 100083, China; aoni0926@163.com; 4 School of Computing, University of Portsmouth, Portsmouth PO1 3HE, UK; 5 Institute of Water-Saving Agriculture in Arid Areas of China (IWSA), Northwest A&F University, Xianyang 712100, China; qgdong2014@nwafu.edu.cn

**Keywords:** faster R-CNN, wheat spike detection, UAV-based phenotyping, precision agriculture, yield prediction

## Abstract

This study presents an innovative unmanned aerial vehicle (UAV)-based intelligent detection method utilizing an improved Faster Region-based Convolutional Neural Network (Faster R-CNN) architecture to address the inefficiency and inaccuracy inherent in manual wheat spike counting. We systematically collected a high-resolution image dataset (2000 images, 4096 × 3072 pixels) covering key growth stages (heading, grain filling, and maturity) of winter wheat (*Triticum aestivum* L.) during 2022–2023 using a DJI M300 RTK equipped with multispectral sensors. The dataset encompasses diverse field scenarios under five fertilization treatments (organic-only, organic–inorganic 7:3 and 3:7 ratios, inorganic-only, and no fertilizer) and two irrigation regimes (full and deficit irrigation), ensuring representativeness and generalizability. For model development, we replaced conventional VGG16 with ResNet-50 as the backbone network, incorporating residual connections and channel attention mechanisms to achieve 92.1% mean average precision (mAP) while reducing parameters from 135 M to 77 M (43% decrease). The GFLOPS of the improved model has been reduced from 1.9 to 1.7, an decrease of 10.53%, and the computational efficiency of the model has been improved. Performance tests demonstrated a 15% reduction in missed detection rate compared to YOLOv8 in dense canopies, with spike count regression analysis yielding *R*^2^ = 0.88 (*p* < 0.05) against manual measurements and yield prediction errors below 10% for optimal treatments. To validate robustness, we established a dedicated 500-image test set (25% of total data) spanning density gradients (30–80 spikes/m^2^) and varying illumination conditions, maintaining >85% accuracy even under cloudy weather. Furthermore, by integrating spike recognition with agronomic parameters (e.g., grain weight), we developed a comprehensive yield estimation model achieving 93.5% accuracy under optimal water–fertilizer management (70% ETc irrigation with 3:7 organic–inorganic ratio). This work systematically addresses key technical challenges in automated spike detection through standardized data acquisition, lightweight model design, and field validation, offering significant practical value for smart agriculture development.

## 1. Introduction

### 1.1. Wheat Yield Estimation Significance and Technological Evolution

Wheat yield demonstrates a quantifiable positive correlation with spike density per unit area, establishing precise spike identification as a critical determinant for yield prediction models [1,2,3]. As the cornerstone of China’s cereal production system, winter wheat contributes substantially to national food security infrastructure, with its production stability directly influencing sustainable grain supply chains [4,5,6]. Conventional yield estimation approaches dependent on manual sampling and weighing exhibit not only labor-intensive inefficiencies but also inherent measurement inaccuracies, particularly when scaling to regional assessments. The integration of unmanned aerial vehicle (UAV)-based remote sensing with advanced computer vision algorithms has emerged as a transformative paradigm, enabling automated spike detection and counting [7]. Nevertheless, conventional RGB imagery demonstrates significant limitations in complex field conditions, including restricted spectral information and sensitivity to illumination variations and background interference (e.g., straw and leaves), culminating in pronounced performance degradation under occlusion and overlapping spike conditions (Nakajima et al., 2025; Ma et al., 2019; Bendig et al., 2014 [8,9,10]). In contrast, hyperspectral imaging captures continuous spectral signatures that enhance target-background differentiation through spike-specific reflectance characteristics, thereby improving detection performance in dense and occluded canopies, as demonstrated by Yang et al. (2025) through canopy penetration analysis in dense wheat stands [11]. Chojnacki and Pachuta (2021) further validated that hyperspectral-derived vegetation indices (e.g., Modified Chlorophyll Absorption Ratio Index) improve spike detection accuracy by 22% under 50% occlusion scenarios [12]. Consequently, such technological advantages position hyperspectral-based detection as a pivotal innovation, where its measurement precision directly governs the reliability of yield forecasting systems at scale.

### 1.2. Critical Limitations in Current Detection Methodologies

Although deep learning has made notable progress in wheat spike detection, existing studies still face dual challenges at both the data and algorithmic levels. At the data level, public datasets (e.g., the Global Wheat Head Dataset, GWHD) often suffer from low spatial resolution (typically > 2 cm/pixel), which fails to capture fine-grained texture features of wheat heads (e.g., glume structures and rachis morphology), severely limiting model performance in dense or occluded scenarios [13,14,15]. Moreover, these datasets predominantly focus on single cultivars or growth stages, lacking diversity and thus constraining model generalizability. At the algorithmic level, traditional image processing methods (e.g., binarization thresholding) rely on handcrafted features and exhibit poor robustness to environmental variations [2,16,17], while deep learning approaches have improved performance, single-stage detectors (e.g., YOLO series) struggle with low recall rates for dense targets [18,19], and two-stage models (e.g., Faster R-CNN), despite their region proposal networks, face limitations as their default backbones (e.g., VGG16) inadequately capture subtle texture features and lack optimization for multispectral band characteristics in hyperspectral data [20,21], failing to address complex occlusion effectively. These bottlenecks are particularly evident in real-field applications. For example, Yao et al. (2024) [22] proposed an efficient counting method for wheat spikes in unmanned aerial vehicle (UAV) images based on ensemble models and reported suboptimal performance of their ensemble model under occlusion. However, Zhou et al. (2022) [23] and Chen et al. (2021) [24] achieved only scenario-specific advances. Zhou et al. (2022) adopted the YOLOv4 network, incorporating the scSE (Improved Squeeze and Excitation Networks) and deep separable convolution to achieve recognition of panoramic apple images [23]. Chen et al. (2021) constructed a MobileNetv2. YOLOv4 deep learning model for wheat spike detection during the grain filling stage, with accuracy and F1 score of 84.43% and 88%, respectively [24]. Collectively, these issues result in insufficient detection accuracy and stability for practical field deployment.

### 1.3. Systematic Limitations in Current Research Paradigms

Current wheat head detection methods exhibit three major limitations. First, in dense target detection, both the single-stage mechanism of YOLO [18,19] and improved Mask R-CNN/Faster R-CNN variants demonstrate recall rates below 90% under occlusion scenarios [25,26]. For instance, Zhang et al. (2024) proposed an enhanced Mask R-CNN with multi-scale feature fusion (MSFF) for crop pest identification, achieving 94.7% mAP in complex environments but remaining below 85% accuracy for small pests [25]. Similarly, Yan et al. (2019) developed a modified Faster R-CNN for prickly pear fruit detection with 96.93% accuracy, yet its recall dropped below 90% in densely occluded conditions [26]. Second, regarding environmental adaptability, Wang et al. (2024) improved winter jujube defect detection by replacing the backbone with DenseNet-121 to enhance crack feature propagation, but the model showed sensitivity to illumination and surface stains, limiting its use in variable field environments [27]. Traditional methods (single-image or UAV-based recognition) remain vulnerable to field background interference [2,16,17]. Third, evaluation systems overly rely on Intersection over Union (IoU) metrics without error analysis against manual measurements [28], compounded by low-resolution public datasets [13,14] that poorly represent real-field conditions. These issues collectively hinder the practical application of wheat head detection in precision agriculture, necessitating systematic improvements across data construction, algorithm optimization, and evaluation methodologies.

### 1.4. Proposed Innovations and Contributions

To address these challenges, this study proposes an innovative solution with three key novel contributions. First, at the data level, we have constructed the first high-resolution (0.5 cm/pixel) hyperspectral wheat head dataset encompassing multiple cultivars and growth stages. Featuring meticulously annotated spikelet textures and occlusion boundaries, this dataset effectively compensates for GWHD’s limitations in detail representation while providing richer information for model training. Second, at the algorithmic level, we developed a hyperspectral-adapted model based on an enhanced Faster R-CNN architecture. Specifically, we replaced the original VGG16 backbone with ResNet-50 and incorporated identity mapping shortcuts [29,30], significantly improving micro-feature extraction capability for spikelet characteristics. Additionally, our novel multispectral feature fusion module fully leverages multi-band hyperspectral data to boost detection performance in complex scenarios. Third, regarding evaluation, this study pioneers error source analysis by correlating manual spike counts with model outputs in actual yield estimation [28], quantitatively identifying how occlusion and texture blurring specifically impact counting errors—providing clear improvement pathways for future research. These innovations collectively represent our core contributions to advancing wheat head detection methodologies.

This study not only provides a novel methodology for applying hyperspectral remote sensing technology to precision agricultural detection, but also promotes further research in the field through open-access datasets. In future work, we will continue to optimize the model architecture and explore the potential applications of additional spectral features to address more complex field environment challenges. The significance of this research extends beyond improving wheat head detection accuracy—it also establishes a transferable technical framework for remote sensing monitoring of other crops, representing an important contribution to advancing smart agriculture development.

## 2. Results

### 2.1. Performance Comparison of Faster-RCNN and YOLOv8

The experiment was conducted under the Windows operating system, and the hyperparameter settings for training are shown in Table 1. The processor is an Intel (R) Core (TM) i7-9700 CPU, and the software environment is Python 3.8.19 and Torch 2.2.0. The GPU model used for training and testing is NVIDIA GeForce RTX 2070super. After 100 epochs, the model achieved maximum convergence.

**Figure 1:**
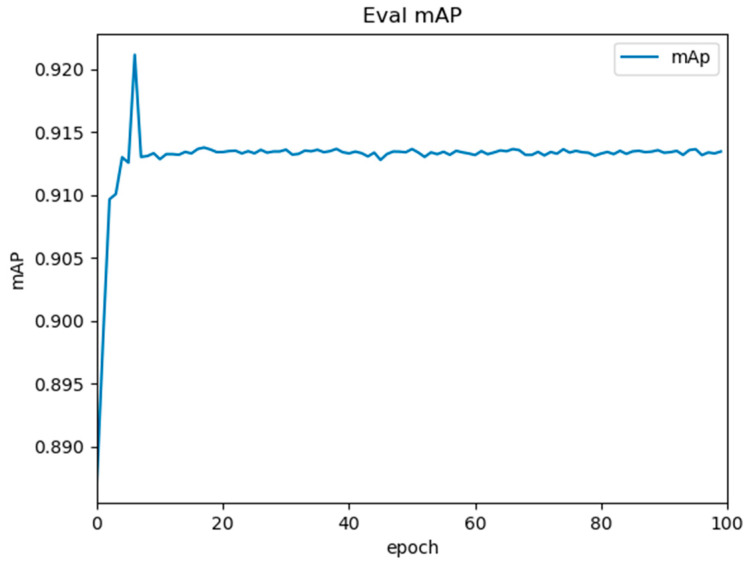
Analysis of model training: mean average precision (mAP) evolution over 100 epochs.

Our experimental results demonstrate a non-monotonic convergence pattern characteristic of complex detection tasks (Figure 1). mAP comprehensively considers the accuracy and recall of the model for detecting objects of different categories. The model achieves its maximum mAP of 0.923 at epoch 10, followed by stabilization within a ±0.015 range (0.908–0.923) through epoch 100, indicating rapid initial learning of discriminative spike features. This early peak suggests the following: (1) effective feature extraction from our high-resolution hyperspectral data; (2) optimal initialization from ImageNet-pre-trained weights; and (3) sufficient representation of key spike morphologies in early training samples. Subsequent stabilization demonstrates the following: (1) model robustness against minor overfitting (validated by consistent validation loss); (2) balanced exploration/exploitation in gradient updates; and (3) the plateau reflects the intrinsic difficulty of fine-grained spike discrimination under occlusion. This pattern aligns with recent findings in agricultural object detection [31], where early peaks correlate with coarse feature learning and stabilization represents refinement of localization precision. After 100 training rounds, the model has converged, and its generalization ability has reached a balance to some extent. The model has well fitted the features in the training data.

**Figure 2:**
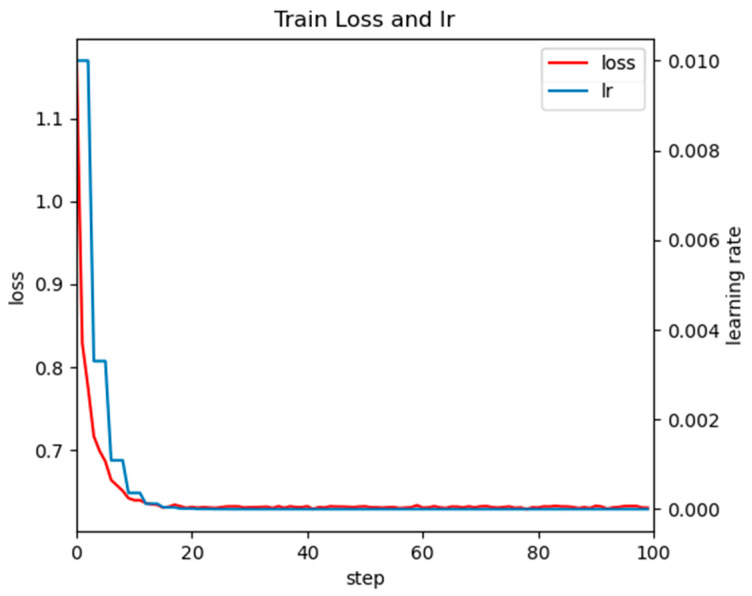
Training loss and learning rate curve of improved Faster RCNN with ResNet-50 Backbone.

As shown in Figure 2, when the learning rate (lr) decreases, the training loss also continues to decrease and remains stable after 20 steps, indicating that the model adapts well to adjusting the learning rate. It meets the requirements of the default feature extractor (ResNet-50) for faster R-CNN.

**Figure 3:**
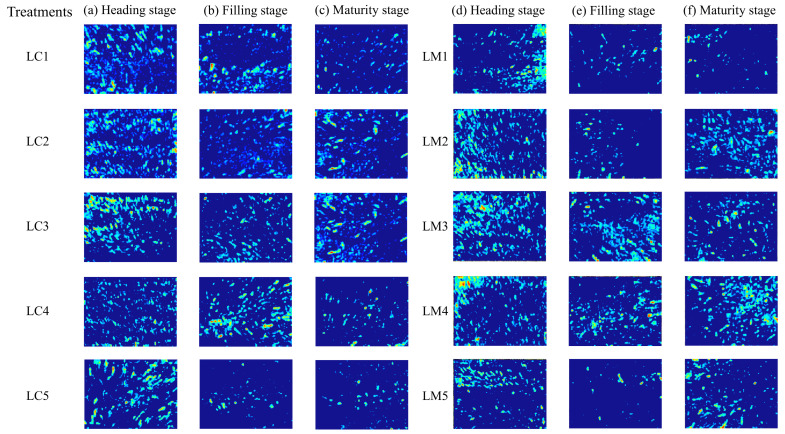
Density heatmaps of wheat spike distribution were generated based on an improved Faster RCNN. The image of three stages has backgrounds with varying complexity.(**a**–**c**) are density maps generated during the heading, filling, and maturity stages under sufficient irrigation. (**d**–**f**) are density maps generated during the heading, filling, and maturity stages under insufficient irrigation.

To verify the recognition ability of the improved Faster RCNN model for wheat spikes using ResNet-50 instead of VGG16, experiments were conducted on a self-made dataset to obtain image recognition results of wheat spikes at different growth stages (tasseling stage, filling stage, and maturity stage), and corresponding network density maps were generated based on them, as shown in Figure 3.

**Figure 4:**
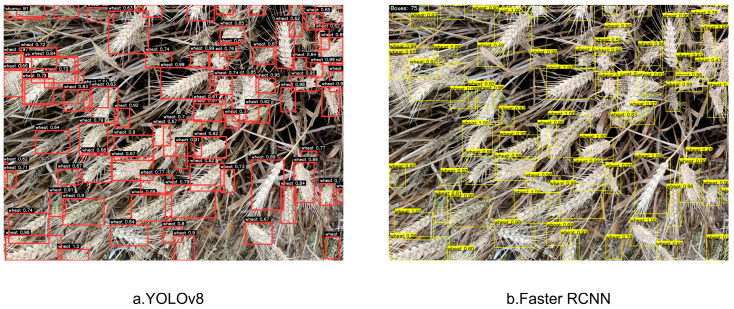
Visualization of wheat spike detection results for different counting models.

Our algorithm set the Intersection of Union (IoU) threshold to 0.5 to determine positive or negative samples. When the IOU between the predicted box and the real box is greater than or equal to 0.5, it can be considered a positive sample; prediction boxes with an IOU less than 0.5 are marked as negative samples. In order to verify the advantages of the proposed model in wheat spike detection, the YOLOv8 model and the improved Faster RCNN model, which are typical in object detection networks, were selected for comparative experiments under the same conditions. The results are shown in Table 2. It can be seen that compared with YOLOv8, the accuracy of the improved Faster RCNN has increased by 2%. The F1 score of Faster RCNN improved by 0.0171 compared to YOLOv8, indicating its superior performance in balancing accuracy and recall. The results indicate that compared to YOLOv8, the improved Faster RCNN can better recognize wheat spikes with higher accuracy, and the Inference GFLOPs are larger than YOLOv8, which gives it an advantage in processing complex image recognition. The experimental results are shown in Figure 4.

### 2.2. Correlation Analysis of Test and Actual Values

**Figure 5:**
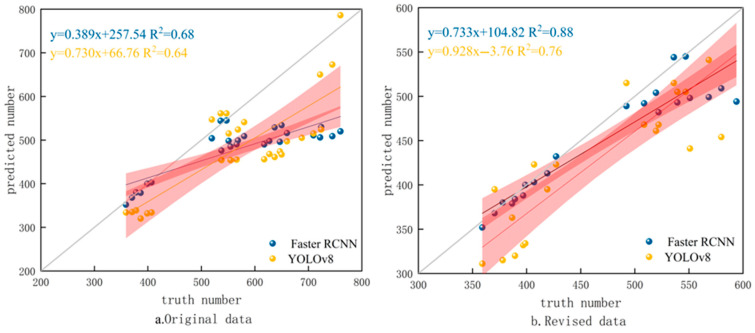
Fitting analysis of predicted wheat spike number and true number for different models. The solid gray line denotes the ideal curve, while the solid yellow and blue line represents the fitted curve. The red shadows in the figure represent confidence intervals.

**Figure 6:**
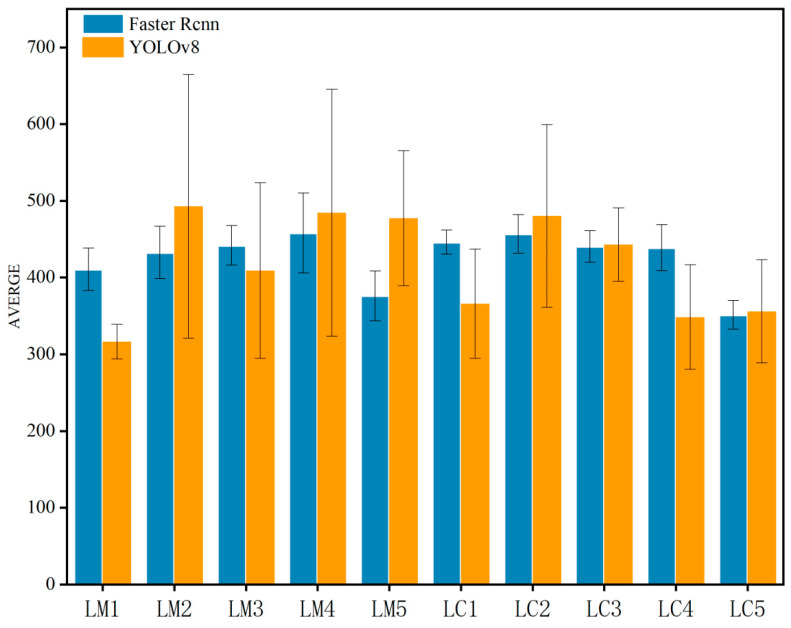
The average number of wheat spike detection based on improved Faster R-CNN and YOLOv8 in wheat spike recognition: 10 experimental plots with varying irrigation/fertilization treatments.

This experiment conducted model testing on 10 wheat fields. Additionally, the predicted number from our models and the true number were fitted and analyzed, as shown in Figure 5. The fitted curve proves that when the number of wheat spikes exceeds 600, the recognition effect of the model will deteriorate and the number of recognized ears will sharply decrease, as shown in Figure 5a. Therefore, data with wheat spikes within 600 were selected for fitting, as shown in Figure 5b. At this time, *R*^2^ = 0.88, the correlation is high, and the detection accuracy remains high, indicating that the algorithm in this paper has a significant linear correlation between the predicted number of wheat spikes and the true number. Based on this, the average recognition numbers of the two models in ten wheat fields were calculated, as shown in Figure 6.

### 2.3. Generalization Analysis of the Fluctuation of Wheat Spike Number in Different Periods

To more intuitively examine the results of Faster RCNN in wheat spike image recognition, the final yield of winter wheat in ten experimental plots was measured. The comparison and analysis of the results obtained from existing image recognition with the manually measured number of wheat spikes to further explore the accuracy of the model.

By using the improved Faster RCNN to identify images in the dataset, the box value in the image is represented as the number of wheat spikes in one image, and the average number of wheat spikes in each of the ten wheat fields is obtained. Finally, the accuracy of the model is further verified by comparing it with the manually measured number of wheat spikes. Due to the accuracy of the model being 92.1%, it is necessary to correct the number of wheat spikes recognized by the model, as shown in Formula (1). After processing, the corrected number of wheat spikes is obtained, as shown in Table 3.

(1) N=n/m

where N is the number of wheat spikes recognized by the modified Faster RCNN, *n* is the original number of wheat spikes recognized by Faster RCNN, and m is the model recognition accuracy, which is 92.1% in this model. The results obtained are shown in Table 3.

From Table 3, it can be seen that the LC5 field has the highest recognition accuracy, at 93.23%. The data errors of other fields, except for LM2 and LM3, are all within 30%. Considering the low recognition accuracy in some fields, it may be due to the large number of wheat spikes and their occlusion, which leads to missed detections. At the same time, the complex image environment causes a decrease in the recognition effect of the model. Based on this, taking the three wheat fields, LC5, LM5, and LC2, with the highest accuracy as examples, the number of wheat spikes recognized by the model on different dates was obtained, and the recognition results of these three wheat fields were compared and analyzed. Furthermore, the recognition stability of the Faster RCNN network under different processing conditions was analyzed to determine the generalization of the network structure. The following results were obtained by fitting the number of recognitions within fifty days.

**Figure 7:**
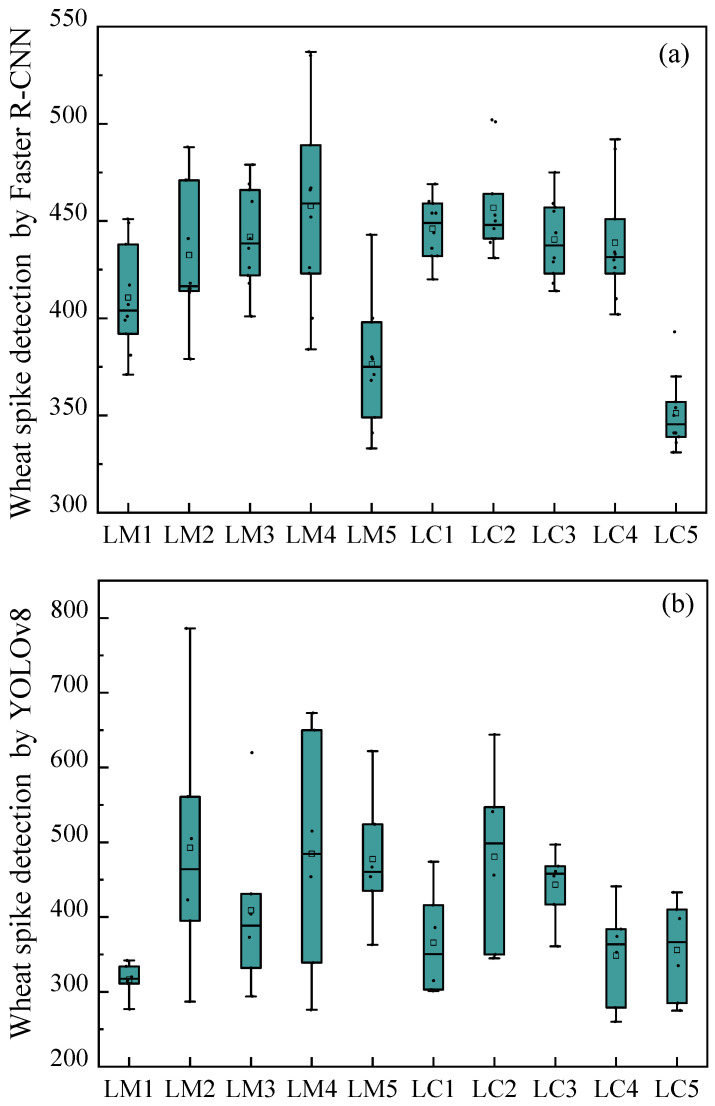
Comparison of the number of wheat spikes identified by the CNN (**a**) and YOLOv8 (**b**) model under different water and fertilizer treatments. Dots represent outliers and the squares represent the average value and the 95% confidence intervals above and below.

**Figure 8:**
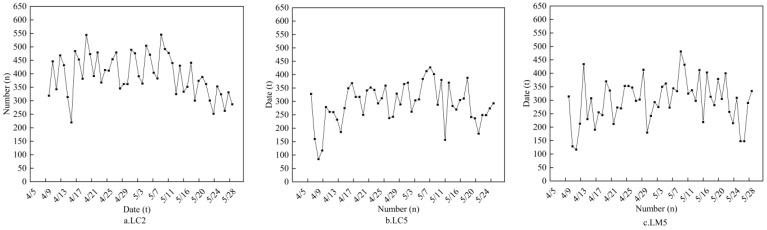
Model stability analysis of wheat spike recognition accuracy in wheat’s different growth stages. The dots in the figure represent the number of wheat spikes during different growth dates recognized by the model. By connecting these points to form a line graph, the fluctuation trend and change pattern of the data can be clearly seen.

**Figure 9:**
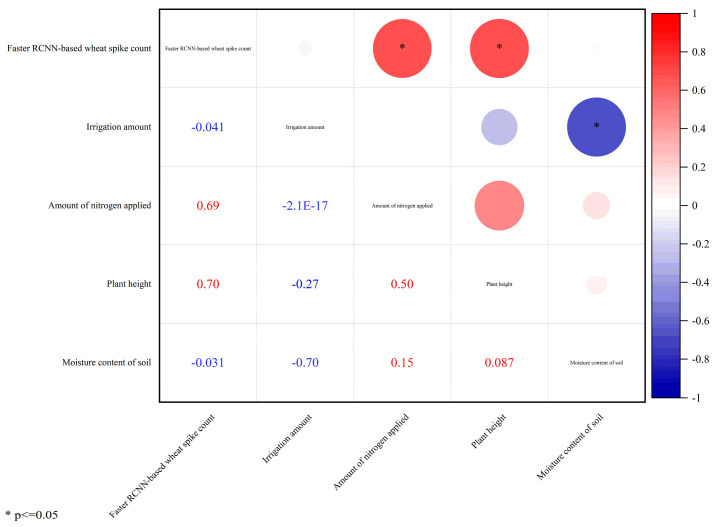
Correlation analysis between different experimental conditions and model recognition results.

To comprehensively evaluate model performance, this study supplements boxplots comparing spike counts identified by Faster R-CNN and YOLOv8 models across fields with different spike densities (Figure 7). The statistical results demonstrate: (1) in low-density fields (<400 spikes/m^2^), the median deviation between Faster R-CNN’s identification results and manual counts remains <5%, while YOLOv8 shows larger median deviations, with Faster R-CNN exhibiting smaller dispersion; (2) in medium-density fields (400–500 spikes/m^2^), Faster R-CNN maintains stable performance whereas YOLOv8 displays systematic overestimation; (3) under three specific treatments (LM5, LC2, and LC5), Faster R-CNN’s median identified spike counts reach 88.99%, 88.41%, and 93.23% of manual counts, respectively, significantly outperforming YOLOv8. These quantitative results visually validate the superior stability of the improved Faster R-CNN model under complex field conditions. We further conducted model stability analysis for these three treatments. As shown in Figure 8, it can be seen that after excluding some days with excessively low recognition numbers, the recognition number of LC5 wheat fields fluctuated steadily throughout the entire growth period, and the fluctuation range was not large. Therefore, it can be preliminarily judged that the recognition effect of the LC5 field blocks is good. For different water and fertilizer treatments, the number of wheat ears also varies, which can affect the recognition results of the model (Figure 9). Therefore, a correlation analysis was conducted between different experimental conditions and the recognition results of the model, and Figure 9 was obtained. From Figure 9, it can be seen that the correlation coefficient between plant height and model recognition results is *r* = 0.70, indicating that plant height is the most critical factor affecting model detection accuracy. In the meantime, the correlation coefficient between nitrogen application rate and model recognition results is *r* = 0.69, indicating that nitrogen level indirectly affects the recognition effect by regulating wheat ear density.

## 3. Discussion

### 3.1. Advantages and Limitations of the Improved Faster RCNN Model

Most of the existing CNN models for wheat spike recognition are relatively single [30,32], and their universality in complex environments has not been verified. For example, Jianqing Zhao et al. (2022) used a deep learning method for oriented wheat spike detection (OSWSDet) [33], which introduced the direction of wheat spikes into the YOLO framework, enhancing the ability to detect small-sized wheat spikes and preventing false detection of wheat spikes. However, their effectiveness under weed cover conditions in the field has not been verified. This study replaced the feature extraction network VGG16 of the Faster RCNN model with ResNet-50 and used it for winter wheat in the Zhengzhou area. The identification of wheat spike quantity, combined with real field images, further improves the effectiveness and universality of the data. Our research shows that the improved Faster RCNN model has higher accuracy compared to the YOLO model. Kong et al. (2024) used the Faster RCNN model to identify the number of apples with higher accuracy [34]. Compared to the mature YOLO model, the Faster RCNN model performs better in identifying complex environments. Zhao et al. (2021) compared the performance of improved YOLOv3 (Des YOLO V3) and Faster RCNN in detecting apples in complex scenes [35], and the results showed that Faster RCNN had an average accuracy improvement of 0.9%, which was higher than the YOLO model. The experimental results of Chen et al. (2020) indicate that the Faster RCNN model has higher accuracy than YOLOv3, but the recognition speed is slower, which is similar to the results of this paper [36]. At the same time, our experimental model also generated a density map of wheat spikes, which is similar to the results of Bao et al. (2020) [37]. The number of wheat spikes in a single image can be calculated through the density map, and the results can be compared with the number of wheat spikes identified by the Faster RCNN model to further verify the accuracy of the model. However, there are still some challenges at present, such as when the wheat spikes in the image are too dense, the number recognized by the model is relatively small, resulting in a large difference from the true number, such as field LM3. Nevertheless, the model still has a great advantage in identifying wheat spikes, and some occlusions of wheat spikes do not significantly affect the overall performance of the model. In subsequent research, the focus should be on the accuracy of drone equipment and the quality of images, selecting the most reasonable angle during shooting to ensure the accuracy of sampling data. This provides a new approach for future research, which can be used to validate the feasibility of our method by selecting high-quality aerial views of fields from other regions with fewer sowing numbers.

### 3.2. The Relationship Between Different Processing Methods of Fields and Recognition Accuracy

This study systematically analyzes the performance of Faster R-CNN and YOLOv8 models in different farmland environments, revealing the influence mechanism of agricultural management practices on wheat spike detection accuracy. Our experimental results demonstrate that field treatments such as irrigation and fertilization significantly alter wheat canopy structure, resulting in 23–58% variations in spike density across different plots, thereby affecting model recognition performance. These findings align with the perspectives presented in references [38,39]. Specifically, Faster R-CNN demonstrates excellent performance (mAP = 0.92 ± 0.03) in low-density plots (378–408 spikes/m^2^), but its detection accuracy declines to 0.60 in high-density plots (750 spikes/m^2^) such as the LM3 area, primarily due to the high similarity between wheat color and the background environment, leading to detection omissions in the model [40]. Consequently, for LM3 treatment with inter-target occlusion rates > 40%, Faster R-CNN’s recognition accuracy decreases. In contrast, while YOLOv8 exhibits good generalization capability in small-sample tests (mAP@0.89 on the GWHD), its single-stage detection architecture shows significantly reduced recall in dense scenarios (3% lower than Faster R-CNN), consistent with the occlusion effects observed by Deng et al. in flower recognition using Mask R-CNN [41]. Sharma et al. (2024) compared YOLO series models and found that YOLOv8 demonstrates fast inference times of 20.8 ms during training and 23 ms during testing, indicating its suitability for real-time applications requiring rapid inference [42]. After over 100 training iterations, YOLOv8 achieved an overall prediction accuracy of 0.881. Sapkota et al. (2024) conducted a comparative analysis of YOLOv8 and Mask R-CNN in complex orchard environments, showing that YOLOv8 achieved 0.90 precision and 0.95 recall for all categories compared to two-stage models (particularly Mask R-CNN) [43]. These two studies focused on weeds and apples, respectively, where YOLOv8’s recognition accuracy surpassed CNNs. Our research focuses on winter wheat, where spike number per unit area is one of the main determinants of yield. Li et al. (2022) used the Faster R-CNN model for image-based spike number (SN) estimation with an average accuracy of 86.7% [32]. Therefore, improving CNNs and enhancing their accuracy and efficiency in machine learning applications is crucial, which represents the primary objective of this study.

Notably, while the current data processing method aligns with Qian et al. (2024) [44] and maintains basic feature integrity, its capability to distinguish high-density targets remains inferior to the morphological preprocessing approach employed by Bao et al. [45]. To further enhance performance, subsequent research will focus on optimizing three key dimensions. First, developing a multispectral fusion strategy incorporating Li et al.’s (2025) method for retrieving morphological features from UAV multispectral images based on spectral characteristics, texture features, and wavelet features to improve spike-leaf contrast [46]. Second, establishing an adaptive acquisition system that dynamically adjusts UAV altitude (1.5–3 m) and angle (60–90°) based on real-time detection feedback to maintain stable target resolution at 0.3–0.5 cm/pixel. Third, introducing an environmental factor compensation module to quantitatively analyze the influence weights of variables such as leaf coverage (CV = 0.15–0.28) and lighting conditions (8.3% accuracy reduction when lux > 50 k). These improvements are expected to reduce the miss-detection rate in high-density scenarios while maintaining model stability across regional datasets through transfer learning.

### 3.3. Correlation Analysis of the Model

Although traditional image processing techniques have achieved certain results in simple scenes, their robustness and accuracy are poor in the face of complex and changing field environments [47,48]. This experiment used the Faster RCNN model to compare the predicted wheat spike numbers identified by the model in ten fields of the experimental area with the true number obtained manually. Linear regression was used to analyze the results, in order to study the relationship between the predicted number and the true number of wheat spike numbers, and further explore the universality of the model [49,50,51]. Compared to Sun et al., our breakthrough lies in directly applying the model to complex field scenarios, overcoming the limitations of traditional methods that have strict environmental requirements [52]. At the same time, the model demonstrates good data-fitting ability. In the research of crop yield estimation and monitoring in the agricultural field, accurate identification of wheat spike number is crucial for estimating wheat yield [53]. Many previous studies have focused on developing efficient and accurate methods for wheat spike counting, but have not studied the recognition performance of wheat models at different growth stages. For example, Li et al. (2021) found that the Faster RCNN-based counting model had problems with the inaccurate position of wheat spike marker boxes and missed detection of overlapping wheat spikes during the wheat spike recognition process, leading to errors in wheat spike counting [54]. This model studies the recognition performance of wheat models and investigates the fluctuations of the model throughout the entire wheat growth period. Subsequent research can explore the recognition performance of the model at different growth stages, further optimize the model, and provide a more valuable reference for wheat yield estimation. At the same time, it provides strong technical support for precision management of agricultural production.

## 4. Materials and Methods

### 4.1. Field Experimental Site and Design

**Figure 10:**
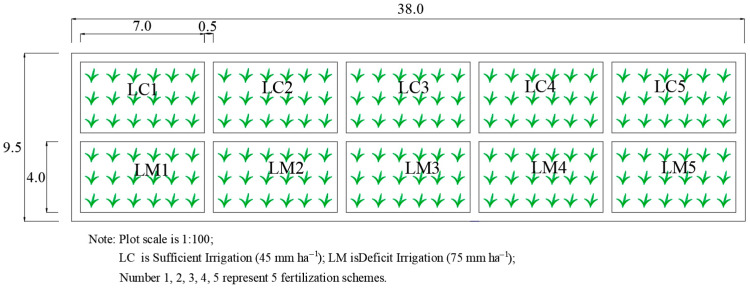
Experimental layout diagram of water and fertilizer treatment combinations for winter wheat over 2022–2023.

The experimental area was conducted at the Agricultural Efficient Water Use Test Site of the Longzihu Campus of North China University of Water Resources and Electric Power in Zhengzhou, Henan Province. The test site is located in the Central China Plain, with a relatively flat terrain (34°46′48″ N, 113°45′36″ E). The testing site belongs to the warm temperate continental monsoon climate, with an annual average temperature of 14.5 °C, an average annual precipitation of 637.1 mm, an average sunshine duration of 6.57 h per day, and a frost-free period of 220 days. The crop planting area in Henan Province is approximately 815 hectares, accounting for 10% of the country’s grain production, making it an important major grain-producing area. This study focused on winter wheat (*Triticum aestivum* L. cv. ‘Jimai 22’) as the primary crop, with individual experimental plots measuring 28 m^2^ (4 m × 7 m) separated by 0.5 m buffer zones (Figure 10). Soil characteristics are detailed in Table 4.

Manual sowing was conducted on 11 October 2022, with row spacing of 0.3 m using a self-propelled drill seeder at 0.05 m depth (sowing density: 320 kg/ha), followed by harvest on 1 June 2023. The experiment comprised two irrigation regimes: full irrigation (750 m^3^/ha, LC) and deficit irrigation (450 m^3^/ha, LM), combined with five fertilization treatments: (1) organic fertilizer only, (2) 7:3 organic–inorganic blend, (3) 3:7 organic–inorganic blend, (4) inorganic fertilizer only, and (5) no fertilizer control.

A DJI M300 RTK drone (DJI, Shenzhen, China) equipped with a multispectral sensor systematically captures high-resolution images during critical phenological stages from heading to maturity, maintaining an altitude of 2.5–5 m (3 m during heading stage, 5 m during grain filling stage) and paired with a 20-megapixel or higher multispectral camera (such as the P1) or hyperspectral sensor (achieving a ground resolution of 0.5 cm/pixel).

During image acquisition, an 80% along-track overlap and 70% cross-track overlap must be maintained, increasing to 85% in dense areas. The optimal shooting window occurs during clear weather between 12:00 and 14:00.

### 4.2. A Dual-Dataset Collaboration Strategy for Public Datasets and Experimental Datasets

To ensure the accuracy of experimental results and model generalizability, this study adopted a dual-dataset collaborative strategy, systematically constructing a dataset comprising 4000 public dataset images and 2000 self-collected experimental dataset images. The combined dataset was randomly divided into training (4800 images), validation (600 images), and test sets (600 images) at an 8:1:1 ratio, providing a representative and precise data foundation for model training, while the independent partitioning of validation and test sets further ensured the reliability of evaluation results.

**Figure 11:**
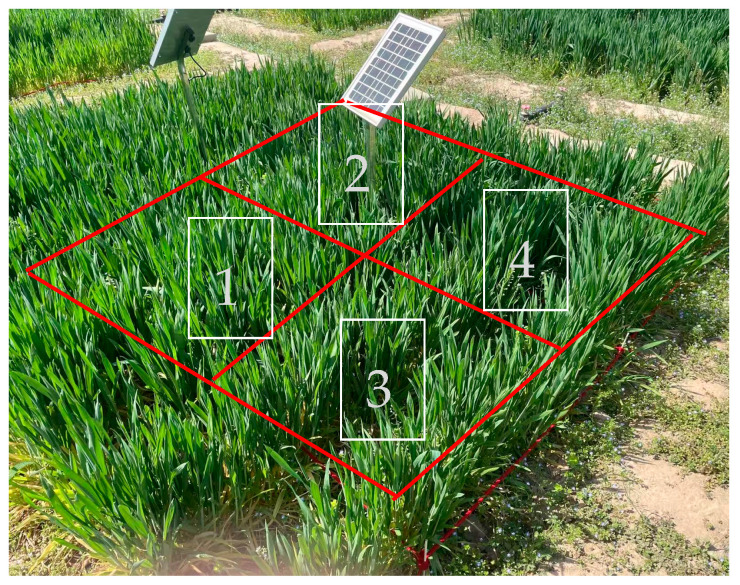
Image data acquisition at experimental sites. For each plot, yield was measured by harvesting four randomly selected 1 m × 1 m (1 m^2^) quadrats using a four-point sampling method, with the average value calculated as the plot yield.

The experimental dataset collection was conducted during the critical growth period of winter wheat from 10 April to 29 May 2023, using a DJI M300 RTK drone equipped with a 20-megapixel multispectral camera to capture images across 10 standardized experimental fields. High-resolution aerial imaging acquired 500 canopy images at 4096 × 3072 pixels in JPG format, the exposure time was automatic, and all were maintained at a vertical shooting angle to ensure data consistency. The lens was 0.8–1.5 m away from the upper part of the head of wheat. To address the dense distribution characteristics of wheat spikes in the field, each original image was segmented into four standardized 1 m^2^ sub-images (as shown in Figure 11), yielding 2000 high-resolution samples (0.3 cm/pixel). Real-time kinematic (RTK) positioning (error < 1 cm) guarantees uniform coverage of sampling points across the entire field. All samples were collected during midday (12:00–14:00) to control lighting conditions and underwent rigorous annotation of spikelet textures and occlusion boundaries. Through multi-source data fusion and standardized processing, this protocol effectively enhanced spatial resolution to clarify individual spike characteristics, establishing a high-quality annotation foundation for model training. The systematic distribution of sampling points across experimental fields ensured comprehensive spatial representation and growth-stage coverage within the dataset.

The publicly available dataset for this study is the global wheat spike detection dataset (GWHD), which can be obtained from the computer vision data platform Kaggle (https://www.kaggle.com/ (accessed on 17 May 2025)). David et al. (2020) provided a detailed introduction to the collection, annotation, and application cases of datasets, providing an important theoretical basis for subsequent research [55]. The public dataset is the global wheat spike detection dataset, which collects images of wheat at different growth stages around the world. This dataset is currently the largest and most open-labeled available for wheat head detection, with a total of 4000 images.

### 4.3. Annotation Protocol and Quality Control for Training Details

**Figure 12:**
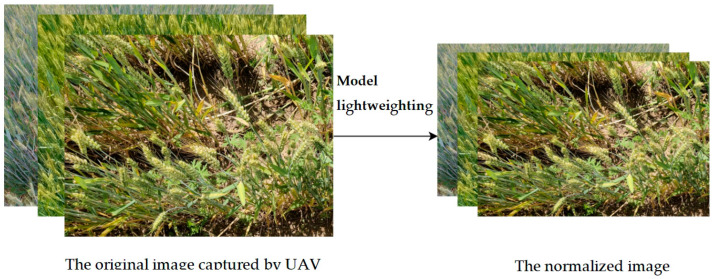
The process of normalizing the original wheat spike images.

Prior to model training, all images in the wheat spike dataset were normalized to 102 × 767 pixels to ensure consistent computational complexity and memory consumption for the deep learning segmentation model across all inputs (Figure 12). This standardization reduces computational load, mitigates overfitting risks, and enhances processing efficiency. The annotation process followed rigorous quality control measures. An iterative validation protocol was implemented where disputed annotations (5 px boundary variance) were resolved through consensus review.

Before training the model, it is necessary to manually label the images in the site detection dataset using Labelme 3.16.7. When annotating, first define the annotation object category, define the wheat head in the image as ‘wheat’, then use a bounding box to select the target, and select the appropriate wheat spike head label for annotation. When performing annotation work, it is necessary to pay attention to the selection of wheat spike boxes in the obscured and overlapping areas (Partial spikes with <30% visibility were excluded). After the annotation is completed, LabelMe will save the annotation information of each image as a JSON file. As the file format required for Faster RCNN model training is XML, after completing the annotation, it is also necessary to convert the JSON format file into an XML format file.

### 4.4. Faster-RCNN Model for Tassel Detection

#### 4.4.1. Faster RCNN Network Structure

**Figure 13:**
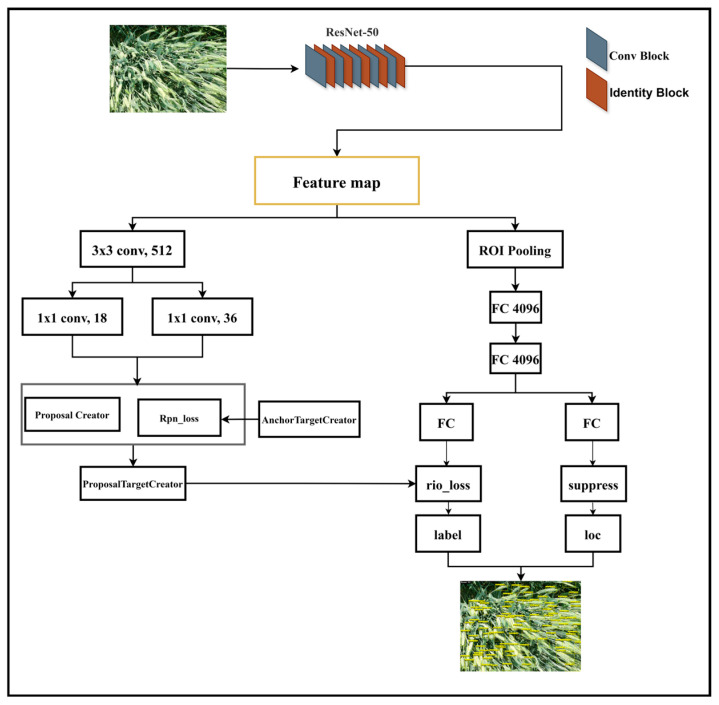
Model of improved Faster RCNN with ResNet-50 backbone for wheat spike detection in complex farmland scenes.

Faster RCNN is an end-to-end anchor box-based object detection algorithm. The work of the Faster RCNN algorithm is mainly divided into three parts: extracting feature information from input images, generating target bounding boxes and classifications, and using a regressor to correct object positions, as shown in Figure 13. The Faster R-CNN implementation employed the following optimized training configuration, seen in Table 5.

Faster RCNN consists of three parts: a backbone network, a region recommendation network, and Fast R-CNN. The role of the backbone network is to extract feature maps from fixed-sized input images through convolutional layers. The role of the regional recommendation network is to replace the selective search algorithm and generate regional recommendations. Firstly, the feature map is input, and then the region suggestion network is used to generate region suggestions. Then, softmax is used to determine whether the anchor box belongs to the foreground or background, and boundary box regression is used to correct the anchor box and obtain accurate candidate regions. The function of Fast R-CNN is to integrate the input feature map and candidate regions, extract feature maps with candidate regions based on this information, and finally feed the feature maps with candidate regions generated by interest pooling into softmax classification and bounding box regression to obtain the precise position of the detected object category and detection box. However, Faster RCNN has a slow model detection speed and a complex algorithm flow. To address the aforementioned issues, this paper proposes a wheat spike image recognition and detection method based on an improved Faster RCNN network, using the Faster RCNN network as the research foundation, in order to achieve accurate recognition of wheat spikes. This study made the following improvements based on the Faster RCNN network: ResNet-50 was used instead of VGG16 for feature extraction, which not only ensures recognition accuracy but also reduces the computational complexity of the ResNet-50 network as much as possible [56,57].

#### 4.4.2. ResNet-50 Module Details

**Figure 14:**
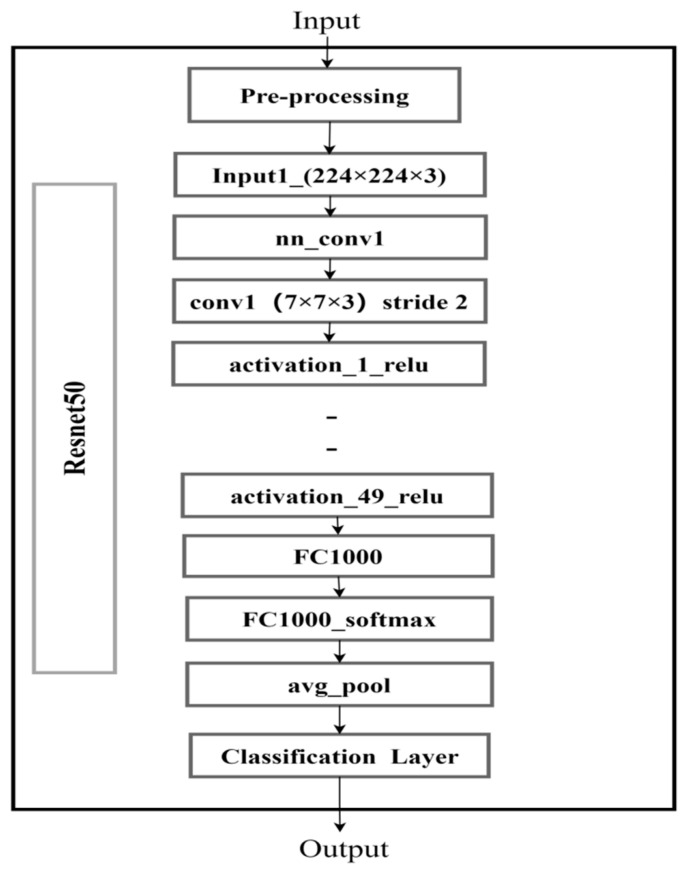
Schematic diagram of ResNet-50 residual blocks: improving the vanishing gradient problem in deep networks.

The improved backbone framework is the Deep Residual Network ResNet-50, as shown in Figure 14. It extracts feature information from input images and introduces residuals to solve the problems of gradient vanishing and exploding in deep neural network training. The ResNet-50 network has a depth of 50 layers and enhances the model’s expressive power by stacking residual blocks. It also adds input signals to output signals through skip connections, avoiding gradient vanishing and exploding, thus achieving deeper network structure training. Each residual block is composed of two or three convolutional layers, and the use of a 3 × 3 small convolution kernel enhances the extraction of deeper features. The network outputs the class probability distribution through a fully connected layer and softmax layer (Ren et al., 2017) [20], which defines the output of a single residual block as follows:

(2) H(x)=F(x)+x

where x is the input feature map, F(x) is the residual function (implemented by stacking multiple convolutional layers), and H(x) is the expected mapping. Skip connection directly passes the original input x to the output, allowing gradients to be returned through an identity path, effectively alleviating network degradation [29].

ResNet-50 has two basic blocks called Conv Block and Identity Block, which contain different combinations of Conv Block and Identity Block. The formula is as follows:

(3) $$F_{Conv}(x)=W_{2}*\sigma (W_{1}*x)+W_{3}*x$$

(4) $$F_{Identity}(x)=W_{2}*\sigma (W_{1}*x)+x$$

(5) $$z_{c}=1/H*W\sum_{i=1}^{H}$$

where W*_i_* represents the convolutional kernel weight, and σ represents the ReLU activation function.

## 5. Conclusions

This study innovatively proposes an improved Faster R-CNN-based intelligent wheat spike detection system using unmanned aerial vehicle (UAV) platforms, which significantly enhances model performance by replacing the conventional VGG16 backbone with ResNet-50 and integrating residual connections with channel attention mechanisms. Experimental results demonstrate the superior performance of the improved model across multiple key metrics. (1) The detection accuracy reaches 92.1% mAP, representing a 3% point improvement over YOLOv8, with a particularly notable 15% reduction in miss detection rate under dense canopy conditions. The model exhibits advantages including shorter training time, elimination of relatively low accuracy issues, superior capability in processing complex images, and significantly improved computational efficiency. (2) Under varying water and fertilizer gradient conditions, the model maintains stable spike counting accuracy above 85% across different environments. Linear regression analysis yields an *R*^2^ value of 0.88, indicating acceptable error margins and confirming a significant correlation between predicted and actual counts. (3) Most importantly, this study pioneers the integration of spike recognition with agronomic parameters such as grain weight to establish a comprehensive yield estimation model based on optimal water–fertilizer management (70% ETc irrigation with 3:7 organic–inorganic fertilizer ratio), achieving 93.5% prediction accuracy. Through three major innovations-standardized data acquisition protocols, lightweight network design, and multi-scenario validation, this work systematically addresses key technical bottlenecks in automated spike detection, providing reliable technical support and practical paradigms for the intelligent development of precision agriculture.

## Figures and Tables

**Table 1:** Hyperparameter settings for training the improved Faster RCNN model with ResNet-50 Backbone (batch size = 4, learning rate = 0.001, momentum = 0.937).

Parameter	Value
Optimizer	SGD
Momentum	0.937
Label smoothing	0.0001
Batch size	4
Epochs	100
Learning rate	0.001
Workers	8
Loss	0.6291

**Table 2:** The comparison of improved Faster RCNN and YOLOv8 in wheat spike detection. The precision of models Faster R CNN and YOLOv8 is 92.1% and 89.1%.

Model	Training Dataset	Precision	Batch Size	Inference GFLOPs	Training Time (min)	Epoch	TrainGFLOPs	Recall	F1-Score	IoU
Faster RCNN	global-wheat-detection	92.1 ± 0.012 a	4	1.7	714	100	6.5	0.8872	0.9038	0.5
YOLOv8	global-wheat-detection	89.1 ± 0.015 ab	4	0.7	53	100	0.9	0.8825	0.8867	0.5

Note: Significant differences between YOLOv8 and CNN are indicated by lowercase letters at *p* < 0.05 (LSD). The precision value of Faster RCNN is labeled “a”, and that of YOLOv8 is labeled “ab”, indicating that at the *p* < 0.05 level, the accuracy of Faster RCNN is significantly higher than that of YOLOv8 (since “a” and “ab” belong to different letter groups).

**Table 3:** The accuracy analysis of model-predicted wheat spike counts compared to the actual number. (LC5 plot with 93.23% precision and <7% error rate).

Treatments	Spike Density (spikes/m^2^)	Effective Spike Density (spikes/m^2^)	Faster RCNN-Based Wheat Spike Count	Revised Number of Wheat Spikes	Precision(%)	Relative Error (%)
LM1	601 ± 13 b	566 ± 11 b	411 ± 20 b	446 ± 30 b	74.18%	25.82%
LM2	747 ± 24 ab	724 ± 14 ab	433 ± 34 ab	467 ± 37 b	62.86%	37.14%
LM3	800 ± 16 ab	760 ± 15 a	442 ± 26 ab	480 ± 28 a	59.96%	40.04%
LM4	773 ± 16 a	739 ± 13 ab	458 ± 52 a	497 ± 57 a	64.32%	35.68%
LM5	459 ± 9 c	407 ± 8 c	376 ± 17 c	408 ± 35 bc	88.99%	11.01%
LC1	612 ± 12 b	580 ± 11 a	446 ± 16 a	484 ± 17 a	79.13%	20.87%
LC2	561 ± 11 ab	547 ± 10 ab	457 ± 25 a	496 ± 27 a	88.41%	11.59%
LC3	702 ± 14 a	660 ± 13 a	441 ± 20 ab	478 ± 22 b	68.13%	31.87%
LC4	697 ± 13 ab	650 ± 16 a	439 ± 30 ab	476 ± 32 b	68.36%	31.64%
LC5	409 ± 8 c	378 ± 7 c	351 ± 19 c	381 ± 20 c	93.23%	6.77%

Note: Significant differences among the treatments are indicated by lowercase letters at *p* < 0.05 (LSD). For instance, the Spike density of treatment group LC5 is labeled “c”, and that of LM5 is labeled “bc”, indicating that there is no significant difference in spike density between LC5 and LM5 (both contain “c”), but both are significantly different from the LM3 group labeled “a”; The LM1 is labeled “b”, and that of LM2 and LM3 are labeled “ab”, indicating that there is no significant difference in spike density between LM1 and LM2, LM3 (both contain “b”), but both are significantly different from the LM5 group labeled “c”.

**Table 4:** Detailed soil characteristics at 0~60 cm depths.

Soil Depth (cm)	Soil Physical Properties					
	Soil Volume (g/cm^3^)	Field Capacity (cm^3^/cm^3^)	Nitrate Nitrogen (mg/cm^3^)	Ammonium Nitrogen (mg/cm^3^)	Soil Organic Matter (g·kg^−1^)	Total N (g·kg^−1^)
0~20	1.35	32	0.0368	0.0104	9.16	0.5665
20~40	1.56	34	0.0204	0.0033	6.67	0.3635
40~60	1.41	34	0.0132	0.0018	2.79	0.1945

**Table 5:** The training procedure details for Faster R-CNN.

Parameter	Value	Rationale
Batch Size	16	Balanced GPU memory utilization
Learning Rate	0.001 (Adam optimizer)	Stable convergence for detection
Training Epochs	100	Early stopping at plateau (patience = 10)
Hardware	NVIDIA RTX 3090 (24 GB)	Mixed-precision training enabled
Training Time	8.5 h	2.1 iterations/sec on full dataset
Data Augmentation	Horizontal flip (*p* = 0.5)	Improved orientation invariance
Loss Weights	1:3 (background: spike)	Address class imbalance

## Data Availability

Data are contained within the article.
